# Structural Characterization of *DDX23* 5′ UTR Regulatory Elements and Their Targeting by LNA-Modified Antisense Oligonucleotides

**DOI:** 10.3390/ijms262211047

**Published:** 2025-11-14

**Authors:** Polina Kamzeeva, Nikita Shepelev, Veronika Zabbarova, Vladimir Brylev, Alexey Chistov, Dmitriy Ryazantsev, Erik Kot, Darya Novopashina, Maria Rubtsova, Andrey Aralov

**Affiliations:** 1 Shemyakin-Ovchinnikov Institute of Bioorganic Chemistry, The Russian Academy of Sciences, 117437 Moscow, Russia; 2 Chemistry Department, Lomonosov Moscow State University, 119234 Moscow, Russia; 3 Institute of Chemical Biology and Fundamental Medicine, Siberian Branch of the Russian Academy of Sciences, 630090 Novosibirsk, Russia; 4 Lopukhin Federal Research and Clinical Center of Physical-Chemical Medicine, Federal Medical Biological Agency, 119435 Moscow, Russia; 5 Educational Resource Center for Cellular Technologies, RUDN University, 117198 Moscow, Russia

**Keywords:** DDX23, hairpin, LNA, antisense oligonucleotides, translation regulation, uORF

## Abstract

Translation of mRNAs is a tightly regulated process in gene expression. In mRNA, the 5′ untranslated region (5′ UTR) controls ribosome recruitment and frequently contains structured elements that modulate translation efficacy. This study investigates stable structural motifs within the 5′ UTR of *DDX23* mRNA, encoding a protein relevant for anticancer therapy, as potential regulators and targets for antisense oligonucleotides (ASOs). Despite bioinformatic predictions and transcriptomic validations suggesting RNA G-quadruplex (rG4) formation, comprehensive structural analysis using a light-up assay and CD, UV, and NMR spectroscopy revealed that most putative rG4-forming sequences do not fold into stable rG4 structures, although one of them exists in an equilibrium between rG4 and an alternative, likely hairpin, conformation. Reporter assays using a robust G4 stabilizer also argue against the significant regulatory role of rG4s in *DDX23* mRNA translation. Instead, we identified and characterized a stable hairpin structure with potential regulatory function. Based on these findings, we designed fully locked nucleic acid (LNA)-modified ASOs to target this hairpin and regions flanking the upstream open reading frame (uORF) and start codon of the coding sequence. A reporter assay demonstrated that cap-proximal targeting achieved robust translation inhibition up to 80%. In contrast, targeting the efficiently translated uORF was ineffective, presumably due to steric hindrances from the ribosomal complex. The study yields crucial design principles for translation-regulating ASOs: avoid targeting regions shielded by efficient uORF translation and carefully tune ASO-RNA duplex stability to surpass endogenous structures without disrupting regulatory mechanisms. These findings provide insights into the regulation of *DDX23* expression and establish a framework for developing ASO-based therapeutics with broad implications for mRNA targeting in anticancer applications.

## 1. Introduction

DDX23, a DEAD-box helicase family member, exhibits versatile regulatory functions in genome stability maintenance, RNA processing and translational control [1,2]. The protein is an integral component of the U5 small nuclear ribonucleoprotein complex, facilitating spliceosomal conformational alterations through RNA duplex unwinding [2,3]. DDX23 maintains genomic stability via R-loop resolution [4] and participates in antiviral immunity through viral dsRNA detection and subsequent IRF3/NF-kB pathway activation [5,6,7].

DDX23 dysregulation is implicated in both cancer development and neurological disorders. Notable upregulation occurs in ovarian, pancreatic, and glial neoplasms, correlating with aggressive phenotypes and unfavorable clinical outcomes. In ovarian cancer, E2F1-driven DDX23 overexpression modulates FOXM1 splicing, promoting oncogenic FOXM1C isoform generation [8]. DDX23 activates the Notch pathway in non-small cell lung cancer, enhancing linc00630 expression [9]. In pancreatic ductal adenocarcinoma, METTL3-mediated m6A methylation stabilizes *DDX23* mRNA, promoting gemcitabine resistance through PI3K/Akt pathway activation [10]. Glioma progression involves DDX23-mediated miR-21 biogenesis regulation [11]. Conversely, DDX23 deficiency or mutations compromise antiviral responses and neurological function, manifesting as developmental delay, autism spectrum disorder, seizures, and dysmorphism [7,12,13]. Current therapeutic approaches targeting DDX23 lack selectivity and include the antiparasitic drug and pan-helicase inhibitor ivermectin, as well as estrogen receptor (ER) antagonists endoxifen and fulvestrant [11,14].

An approach for modulating gene expression with high specificity and effectiveness explores antisense oligonucleotides (ASOs) that are designed to bind to specific RNA sequences. A number of ASOs have been approved for commercial use [15], and they are increasingly recognized as powerful tools for uncovering the biological functions of genes. The main antisense mechanisms include RNase H1-dependent mRNA degradation and pre-mRNA splicing regulation for down- and up-regulation of gene expression, respectively [16]. However, ASOs can also utilize other mechanisms, such as translation inhibition due to steric hindrance or no-go decay degradation [17,18,19]. To increase gene expression, ASOs are designed to disrupt mRNA 5′ UTR secondary structures or mask upstream open reading frames (uORF) that negatively affect translation of the coding sequence (CDS) [20,21].

Chemical modifications of ASOs have significantly enhanced their therapeutic potential through the improvement of target hybridization, stability, and cellular uptake. Striking examples include phosphorothioate (PS) backbones, 2′-*O*-methoxyethyl (2′-MOE) nucleotides, and locked nucleic acids (LNAs), which have proven effective in the treatment of genetic diseases [15]. LNAs represent ribonucleotide analogs featuring a methylene bridge between the 2′-O and 4′-C atoms of the ribose ring [22]. This structural constraint favors a 3′-endo conformation of the ribose moiety that results in optimized hybridization characteristics and a context-dependent increase in melting temperature (T_m_) up to 10 °C per modified nucleotide [23]. Additionally, LNA incorporation at ASO termini can enhance nuclease resistance, thus improving their therapeutic efficacy.

ASOs targeting 5′ UTR can interact with regulatory structural elements, such as hairpins and G-quadruplexes (rG4s) [21,24]. rG4 structures can fold within guanine (G)-rich sequences and consist of stacked G-quartets stabilized by a network of Hoogsteen hydrogen bonds and monovalent cations such as K^+^ [25]. In most cases, hairpins and rG4s function as translation inhibitors by acting as steric hindrances to the scanning pre-initiation complex (PIC) [25,26,27]. Previous transcriptomic analyses and bioinformatic predictions have indicated that *DDX23* mRNA 5′ UTR contains putative rG4s [28,29]. Furthermore, RNA fold analysis suggests the presence of hairpin structures within the 5′ UTR [30,31]. Here, we employ spectroscopic analysis and LNA-based functional probing to characterize these structural elements and evaluate their regulatory capacity and targetability using a dual luciferase assay.

## 2. Results and Discussion

### 2.1. Analysis of Potential Regulatory G-Quadruplex Elements in 5′ UTR of DDX23 mRNA

The rG4 structure was previously identified in the ENST00000551468 transcript (GENCODE release 26) of *DDX23* during investigation of its translation regulation by predominately rG4-resolving DEAH-box helicase DHX36 [28]. Although this finding is important, the older and the more recent annotation (GENCODE release 48) imply that this specific isoform has a truncated 3′ end without the full coding sequence and 3′ UTR. Moreover, the sequence of the studied rG4 does not map to any other transcripts. Therefore, this rG4 has no influence on the isoforms coding full-length DDX23 proteoforms. Thus, we searched for rG4s in the MANE Select [32] isoform of *DDX23* (ENST00000308025), which is more functionally relevant.

**Figure 1:**
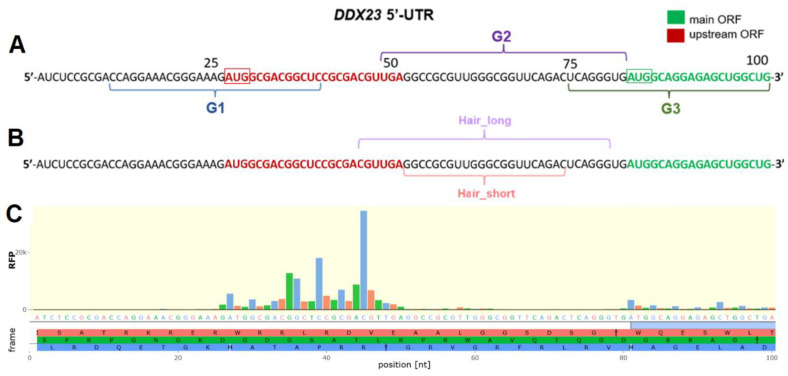
Putative rG4- and hairpin-prone sequences within 5′ UTR of *DDX23* mRNA. RNA sequences G1, G2, and G3 that contain rG4-forming motifs predicted by QGRS mapper and 3-nt flanks (**A**) and hairpin-forming RNA sequences Hair_short and Hair_long predicted by RNA fold [30,31] (**B**). Aggregated ribosome profiling data are shown using Ribocrypt and are divided into three reading frames, which are colored red, green, and blue. The corresponding amino acids and stop codons (asterisks) are shown in reading frames. AUG codons and stop codons are marked with white or black vertical bars, respectively (**C**). RFP—ribosome footprints, nt—nucleotide. 5′ UTR and the proximal region of the coding sequence of *DDX23* mRNA are shown.

Computational analysis using QGRS software (https://bioinformatics.ramapo.edu/QGRS/index.php, accessed on 22 May 2025) identified putative rG4-forming motifs within the *DDX23* mRNA 5′ UTR exclusively (14–36 nt and 51–77 nt) and its extended variant including the proximal region of the coding sequence (14–36 nt and 76–96 nt) [33]. Transcriptomic techniques DMS-seq and G4RP-seq corroborated the formation of rG4 structures within 14–36 nt and 76–96 nt sequences (Figure S1) [29,34,35]. To study the putative rG4-folding in vitro, we synthesized RNA sequences that contain these rG4-forming motifs and 3-nt flanks, as well as control mutants with guanine-to-uridine substitutions (Figure 1A, Table S1): G1 (11–39 nt), G2 (48–80 nt), and G3 (73–99 nt).

**Figure 2:**
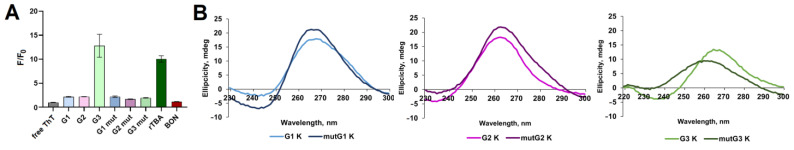
Characterization of putative rG4-forming sequences using fluorescence and CD spectroscopy. ThT-based light-up assay (**A**). The ThT fluorescence intensity (F) was monitored at 485 nm upon excitation at 440 nm. Conditions: 2 µM RNA, 1 µM ThT, 20 mM sodium phosphate buffer, pH 7.4, 140 mM KCl, 25 °C. CD spectra of putative rG4-forming sequences G1, G2, and G3 and their G-to-U mutants mutG1, mutG2, and mutG3 (**B**). Conditions: 2 µM RNA, 20 mM sodium phosphate buffer, pH 7.4, 140 mM KCl, 15 °C.

We began the structural characterization of the sequences with a thioflavin T (ThT)-based light-up assay [36] (Figure 2A). rTBA and BON were used as the control rG4 and RNA duplex, respectively (Table S1) [37]. The CD spectrum of rTBA with a positive peak at 260 nm and a negative peak at 238 nm corresponded to a parallel rG4 structure, while the presence of a positive peak at 265 nm in the BON CD spectrum was attributed to a hairpin one (Figure S2A). Only the G3 sequence exhibited an enhancement in fluorescence intensity comparable to control rTBA, which suggests rG4 formation.

Since the results of the ThT light-up assay usually require additional validation due to possible false-positive outcomes [38], circular dichroism (CD) spectroscopy was used for additional structural analysis (Figure 2B). rG4s are typically parallel-stranded [26] and display characteristic CD spectra with positive and negative peaks at 263–267 nm and 240 nm, respectively [39,40,41]. However, the characteristic signals for RNA duplexes also appear in the same spectral region, typically with a maximum at 260–270 nm and with or without a minimum at 230–240 nm [42,43,44,45]. This complicates the unambiguous assignment of secondary structure. The CD signal pattern of G3, with a maximum at 265 nm and a minimum at 237 nm, is most consistent with rG4. Furthermore, only the G-to-U mutant of G3 exhibited a decrease in ellipticity. This again indicates that G3 is the most probable rG4-forming sequence. For G1 and G2, the ellipticity of the mutants was higher, which reinforces doubts about their ability to form G4s, and we did not observe perfect alignment of signals with the characteristic peaks of rG4. Thus, among the three sequences, G3 has the highest probability of forming the rG4 structure; however, the slight increase in ellipticity compared to the mutant cast doubt on this conclusion and necessitate further verification.

Comparative CD analysis of native and mutated sequences was performed in potassium and lithium-containing buffers (Figure S3) [46,47]. K^+^ cations typically stabilize rG4 structures by coordinating the guanine O6 atoms within G-quartet, whereas Li^+^ cannot provide such stabilization due to its larger hydrated radius and the higher energy cost of dehydration [48,49]. Only G3 exhibited modest spectral alterations upon K^+^-to-Li^+^ replacement, with peaks shifting towards characteristics of RNA A-form helix (positive and negative peaks at 268 nm and 235 nm, respectively). The minimal spectral changes observed for all other sequences, both wild type and mutant, further supported the inability of the G1 and G2 sequences to fold into rG4 structures.

**Figure 3:**
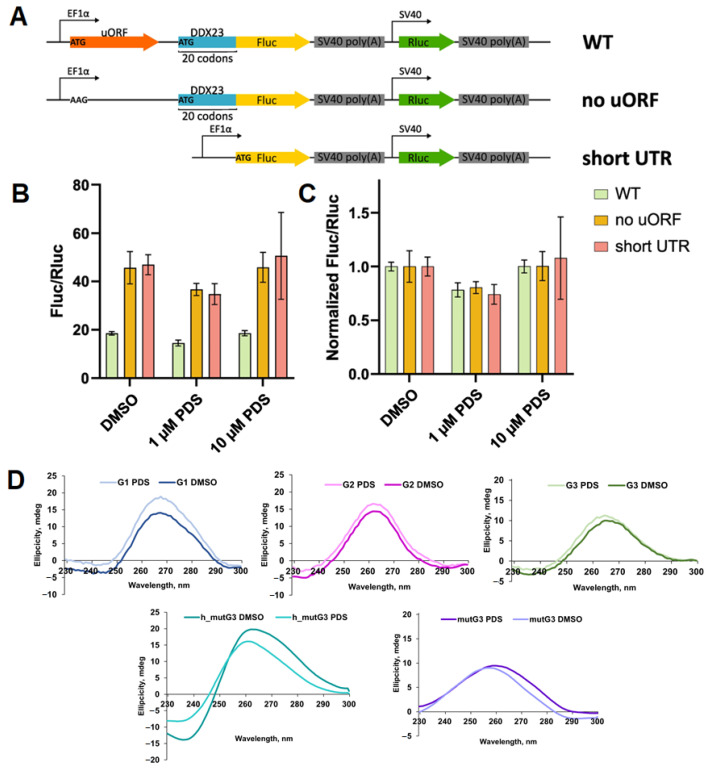
Effect of PDS treatment on relative luciferase activities for reporter plasmids and CD spectra of putative rG4s. Plasmids encoding native *DDX23* mRNA 5′ UTR (WT), *DDX23* mRNA 5′ UTR with mutation in the uORF start codon (no uORF), and control short 5′ UTR lacking rG4s-prone motifs and uORF (short UTR) were used (**A**). Ratios of Fluc and Rluc activities for the reporter constructs tested (**B**). Normalized ratios of Fluc and Rluc activities to DMSO control (**C**). Means and standard deviations are shown. CD spectra of putative rG4-forming sequences G1, G2, and G3, as well as rG4-forming h_mutG3 and hairpin-forming mutG3, in the presence of 1 eq. PDS or DMSO as a control (**D**). Conditions: 2 µM RNA, 20 mM sodium phosphate buffer, pH 7.4, 140 mM KCl, 15 °C.

The results obtained for G3 in vitro led us to investigate whether it influences the *DDX23* mRNA translation when treated with pyridostatin (PDS), a potent G4 stabilizer [50]. Notably, rG4 structures positioned downstream of uORFs may enhance uORF translation, thus suppressing protein synthesis from CDS through synergistic inhibition [28]. To evaluate the impact of G3 stabilization, if formed in cellulo, a dual luciferase assay was conducted (Figure 3). The reporter plasmid encodes *Renilla* luciferase (Rluc) as a control as well as the native 5′ terminus of *DDX23* mRNA, which is fused to firefly luciferase (Fluc) without AUG codon. Additionally, we utilized the same construct lacking the uORF start codon, in which ATG was mutated to AAG, and a construct with a different short 5′ UTR lacking rG4-forming motifs and uORFs (Figure 3A).

We observed that uORF induces approximately 2.5-fold translation repression, as accessed by the change in the Fluc/Rluc ratio following uORF inactivation regardless of the presence of PDS or DMSO (Figure 3B). This result aligns with efficient translation of uORF according to ribosome profiling data (Figure 1C). At the same time, PDS treatment induced a similar change in relative luciferase activities across all constructs tested (as evident after normalization to DMSO), calling into question the presence of rG4 or its influence on translation (Figure 3C).

Given the results of luciferase reporters, additional CD spectra were recorded in the presence of PDS for the sequences under study (Figure 3D) and the control rG4 and RNA duplex (Figure S2B). A significant alteration in CD ellipticity was observed only for G1, while for G2 and G3, the changes were minimal. However, considering previous experimental results, rG4 formation in the G1 sequence remains unlikely. The absence of the G3 response to the PDS treatment contradicted rG4 folding, despite the ThT-based light-up assay providing encouraging results and the CD spectral changes during K^+^-to-Li^+^ substitution (Figure 2A and Figure S3).

**Figure 4:**
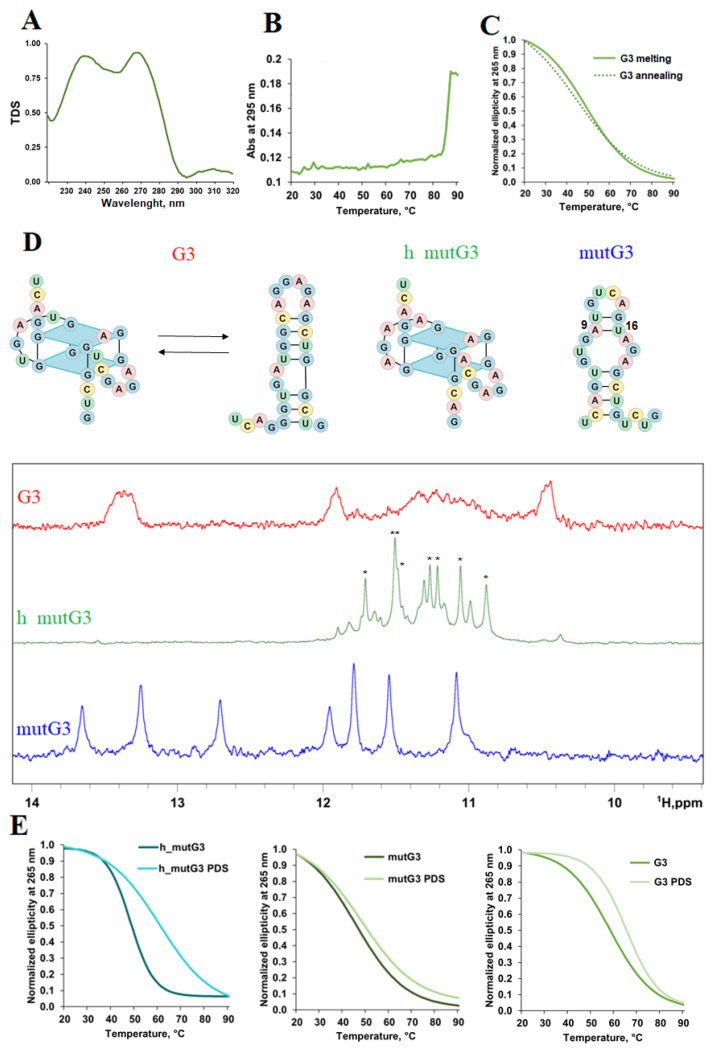
Spectral characterization of G3 and its rG4- and hairpin-forming mutants h_mutG3 and mutG3, respectively. TDS spectra of G3 (**A**). UV melting curves registered at 295 nm for G3 (**B**). CD melting and annealing curves registered at 265 nm for G3 (**C**). Proposed secondary structures and exchangeable protons region (9.0 to 14.0 ppm) of ^1^H NMR spectra for G3, h_mutG3, and mutG3 (**D**). * indicates signals from the guanine imino protons of the main h_mutG3 conformer. CD melting curves registered at 265 nm for G3, h_mutG3, and mutG3 alone or in the presence of 1 eq. PDS (**E**). Conditions for A–C, E: 2 µM RNA, 20 mM sodium phosphate buffer, pH 7.4, 140 mM KCl; for D: 100 μM RNA, 25 mM PBS, 100 mM KCl, 90:10 H_2_O/D_2_O, pH 7.0, 25 °C.

For further structural validation of G3 folding pattern, thermal difference spectra (TDS) and UV-melting analyses were used [51,52]. These methods revealed no characteristic hypochromic shift at 295 nm during melting, typically associated with rG4 structures, although TDS displayed a minor negative peak around 295 nm (Figure 4A,B).

We hypothesized that G3 may fold into an alternative structure such as a hairpin. The potential hairpin formation is supported by the sequence’s capacity to form three stable canonical GC pairs and four GU wobble pairs [30,31,53]. CD melting of G3 was monophasic with T_m_ of 58 °C, indicating sufficient stability of the folded structure under physiological conditions (Figure 4C). Minimal hysteresis suggests an intramolecular structure [54].

The G3 structure was then investigated using ^1^H NMR spectroscopy (Figure 4D). A broad signal in the 10.8–11.8 ppm region may indicate rG4 formation, whereas other broad signals at 10.4, 11.9, and 13.4 ppm may correspond to the imino protons engaged in Watson–Crick base pairing. A poor signal-to-noise ratio, possibly due to aggregation or interconversion, prevented unambiguous structural assignment. The comparative analysis was conducted using two mutant sequences: mutG3 (a mutant sequence with no ability to form rG4) and h_mutG3 (designed to form exclusively rG4 structure through substitution of wobble pair-forming uridines with adenines) (Table S1). ^1^H NMR spectra of both mutants yielded clear interpretable signals. The sequence h_mutG3 demonstrated formation of a two-tetrad rG4, evidenced by eight signals from guanine imino atoms in the 10.8–11.8 ppm region, with minor fraction of an alternative rG4 conformation (Figure 4D). The mutG3 spectrum corresponded to the RNA hairpin-like conformation and contained four signals from imino protons of GU wobble pairs and three signals from canonical AU and GC pairs (Figure 4D). The absence of the eighth signal from imino protons can be explained by the high positional entropy of U16 within the A9-U16 pair (according to the RNAfold server, http://rna.tbi.univie.ac.at//cgi-bin/RNAWebSuite/RNAfold.cgi?PAGE=3&ID=Yf9xnNJuKt, accessed on 2 September 2025), suggesting that this residue is involved in many different pairing states [55]. Comparative analysis of all three spectra suggests that G3 may form both rG4 and hairpin structures, as its ^1^H NMR spectrum contains imino proton signals in regions characteristic of both rG4 (as in h_mutG3) and Watson–Crick GC base pairs (as in mutG3).

CD spectra of mutG3 and h_mutG3 corroborated the structural assignments derived from the ^1^H NMR experiments. The CD spectrum of mutG3 had a positive peak at 260 nm, without a minimum at 240 nm, which corresponds to a hairpin structure [42,56] (Figure S4). The presence of a positive peak at 263 nm and a negative peak at 240 nm indicated that h_mutG3 was folded into the rG4 structure. The addition of PDS did not affect the CD ellipticity of mutG3, as was the case for the control RNA duplex BON but resulted in its decrease for h_mutG3, similar to the decrease observed for the control rG4 rTBA (Figure 3D and Figure S2B). According to CD melting experiments, h_mutG3 was stabilized in the presence of 1 eq. PDS by 12 °C, while moderate (+7 °C) and little (+3 °C) changes in T_m_ for G3 and mutG3, respectively, were revealed (Figure 4E). The observed increase in G3 T_m_ does not contradict the absence of an increase in the CD maximum (Figure 3D), as has already been demonstrated for G4 ligands, including PDS [57,58]. In addition, UV melting proven to be ineffective for studying small RNAs that fold into alternative metastable structures [59]. These results confirmed that G3 could fold, at least partially, into rG4 in vitro.

To summarize this part, while the G3 sequence may adopt hairpin-like and rG4 conformations at equilibrium in vitro, as confirmed by spectroscopic methods, cell-based assays showed no response to the treatment with the G4 stabilizer PDS. Thus, none of the selected sequences forms a stable rG4 structure that may be regulatory. In contrast, a hairpin can be formed within the G3 sequence or the other part of the 5′ UTR. Such hairpin(s) could be involved in the regulation of *DDX23* mRNA translation.

### 2.2. Analysis of Regulatory Stem-Loop Elements

**Figure 5:**
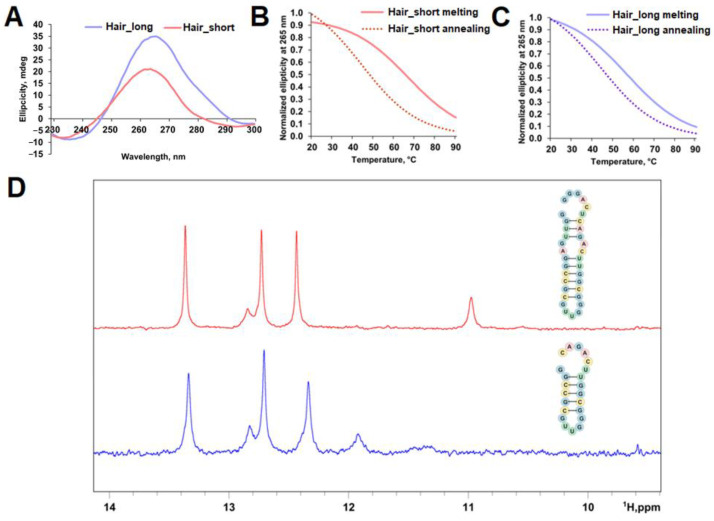
Spectral characterization of Hair_long and Hair_short. CD spectra of Hair_long and Hair_short (**A**). Conditions: 2 µM RNA, 20 mM sodium phosphate buffer, pH 7.4, 140 mM KCl, 15 °C. CD melting and annealing curves registered at 265 nm for Hair_short (**B**) and Hair_long (**C**). Conditions: 2 µM RNA, 20 mM sodium phosphate buffer, pH 7.4, 140 mM KCl. Exchangeable protons region (9.0 to 14.0 ppm) of ^1^H NMR spectra for Hair_long (red line) and Hair_short (blue line) (**D**). Conditions: 100 μM RNA, 25 mM PBS, 100 mM KCl, 90:10 H_2_O/D_2_O, pH 7.0, 25 °C.

We identified the most probable hairpin within the 44–64 nt region by RNA fold [30,31]. Two oligoribonucleotides were synthesized to evaluate the influence of sequence context: Hair_short (minimal hairpin-forming sequence) and Hair_long (minimal hairpin-forming sequence with 6-nt flanks) (Table S1, Figure 1B). CD spectra showed maxima at 265 nm and minima at 233 nm (Hair_short) and 236 nm (Hair_long), consistent with A-form RNA helix formation (Figure 5A). CD melting analysis revealed a T_m_ of 56 °C for Hair_long and 66 °C for Hair_short, indicating physiological stability despite the negative impact of additional 6-nt flanks on T_m_ (Figure 5B,C). Conditions with slow annealing produced alternative structures with uniform melting temperatures of 44 °C for both constructs (Figure 5B,C).

^1^H NMR spectra of both sequences contained three distinct signals that were attributed to guanine imino protons from Watson–Crick GC pairs (Figure 5D). Thus, CD and ^1^H NMR spectroscopies confirmed the hairpin structure predicted by RNA fold [30,31], which should be thermodynamically stable under cellular conditions.

The identified structured elements within 44–64 nt (Hair_short) and 73–99 nt (G3) regions of *DDX23* mRNA 5′ UTR may act as a translation-inhibiting element. While the thermal stability of Hair_short appears insufficient for substantial translation inhibition (T_m_ around 60 °C, less than 13 kcal/mol based on ViennaRNAfold computational analysis [30,31]), its position downstream of the uORF start codon might facilitate synergistic inhibition of CDS translation, consistent with previous observations in other systems and partially explaining efficient uORF translation indicated by ribosome profiling data (Figure 1C) [60,61,62]. In turn, G3 also has a T_m_ close to 60 °C and may promote the recognition of the uORF start codon and inhibit CDS translation.

### 2.3. Evaluation of LNA-Based ASOs Activity in DDX23 5′ UTR Translation Control

**Figure 6:**
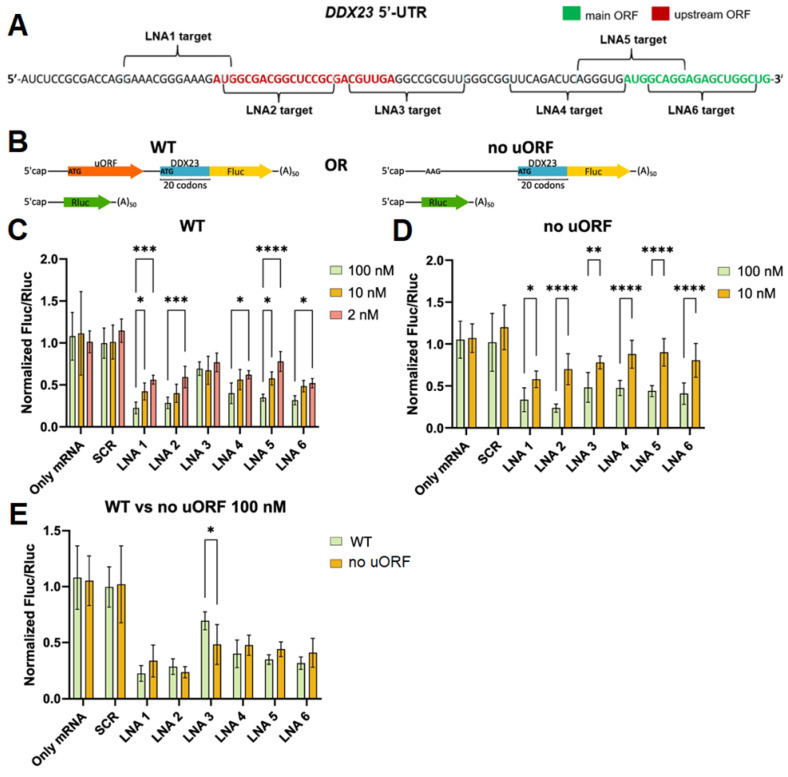
Design and effects of LNA-ASOs at different concentrations used on translation efficiency of mRNA containing the *DDX23* 5′ UTR. Designed ASOs targeting structured and/or putative regulatory elements (**A**). 5′ UTR and a proximal region of the coding sequence of *DDX23* mRNA are shown. Schemes of the reporter mRNA used (**B**). Results for the wild-type 5′ UTR (**C**), 5′ UTR with point substitution with no uORF (**D**). Comparison of results for wild-type and no uORF constructs at 100 nM LNA concentrations (**E**). Means and standard deviations are shown. For accurate normalization, Fluc/Rluc ratios were divided by the median of only mRNA samples for each condition. Ordinary two-way ANOVA with Šidák correction for multiple comparisons were performed. *—adjusted *p*-value < 0.05, **—adjusted *p*-value < 0.01, ***—adjusted *p*-value < 0.001, ****—adjusted *p*-value < 0.0001.

Since downregulation of *DDX23* expression has shown therapeutic potential in cancer treatment, and current approaches are limited to non-selective helicase inhibitors and siRNAs with a transient silencing effect [8,11], we designed a set of fully LNA-modified ASOs targeting start codons of uORF and CDS and/or the structured regions Hair_short and G3 within *DDX23* mRNA 5′ UTR and assessed their effects on the translation efficacy (Figure 6A, Table 1). LNA modifications were used to enhance nuclease resistance and hybridization properties, the latter being particularly important for unfolding of stem-loop structures. Although fully LNA-modified ASOs cannot activate RNase H-induced cleavage [63], a previous study demonstrated that such ASOs targeting 5′ UTR can equally or, in some cases, more effectively suppress mRNA translation compared to LNA-DNA chimeras with predicted ability to recruit RNase H [64]. Furthermore, fully modified blocker ASO targeting 5′ UTR can enhance the efficacy of RNase H1-activating ASOs that target mRNA coding regions [65].

Several LNA-modified ASOs inevitably occlude the start codon of the uORF and/or disrupt the secondary structures previously identified in our analysis, which could theoretically promote translational activation. Nevertheless, fully LNA-modified ASOs are anticipated to generate highly stable duplexes with the target mRNA in close proximity to the 5′ cap—exhibiting greater thermodynamic stability than native structural elements—thereby predictably suppressing translation initiation. According to the literature, translational enhancement is achieved exclusively through site-specific incorporation of constrained ethyl (cEt) modifications, which are structurally akin to LNA, not fully LNA or cEt-modified ASOs [20,21].

Since the efficiency of ASO translation inhibition depends on the stability of the ASO-RNA duplex, T_m_ was calculated using a T_m_ predictor (Table 1) [66]. The predictor data showed that all LNAs form duplexes with high stability under physiological conditions, exceeding the stability of the endogenous structures that were detected in the 5′ UTR (with T_m_ < 60 °C). Therefore, LNAs were predicted to be highly effective in binding to mRNA and inhibiting translation. To evaluate LNA-modified ASO efficacy, we performed a dual luciferase assay using mRNA-based reporters to avoid effects associated with transcription, splicing, and nuclear export and to use precise 5′ UTR sequences [67]. Fluc mRNA was synthesized using the wild-type DDX23 luciferase reporter construct and the construct with the inactivated uORF start codon (Figure 6B). For the control, we synthesized Rluc mRNA (Figure 6B) (see Section 3). HEK293T cells were co-transfected with Fluc reporter and Rluc control mRNAs as well as with ASOs LNA1-6 or the scramble LNA (SCR). The ASOs were used at 2, 10, and 100 nM concentrations, with SCR demonstrating no significant effects on the Fluc/Rluc ratio at any concentration compared to no ASO control (Figure 6C,D).

We observed translational repression with all ASOs targeting the *DDX23* reporter mRNA of the wild type, particularly at a 100 nM concentration (Figure 6C). This result may be explained by the general inhibition of the translational machinery’s activity on the reporter mRNA. Nevertheless, the effects were not uniform across ASO types and concentrations. ASOs LNA1 and LNA2 target regions around the uORF start codon and should therefore mask it and repress uORF translation while enhancing CDS translation. However, they were the most effective inhibitors among ASOs under study at 10 and 100 nM concentrations (Figure 6C). This may be explained by their cap proximity, which hinders PIC loading. Interestingly, LNA ASOs exhibited a concentration dependency except for LNA3, which was a less effective inhibitor. This difference was especially noticeable at 100 nM concentration (Figure 6C). Such an effect may be explained by the targeting of uORF or Hair_short. To resolve this issue, we performed the same experiment with the *DDX23* reporter mRNA lacking the uORF start codon. All LNAs showed inhibitory effects at 10 and 100 nM concentrations, which were concentration-dependent for all LNA ASOs (Figure 6D).

All LNA-modified ASOs except LNA2 and LNA3 demonstrated marginally diminished inhibitory effects for the no uORF reporter in comparison to the wild-type mRNA reporter (Figure 6E). Weaker effects observed for LNA2 in the mutated construct may be attributed to a nucleotide mismatch at its 3′ end because of the point substitution in the uORF start codon. Such mismatch should result in a reduction of the thermodynamic stability of the RNA–LNA duplex and also make it a more favorable substrate for RNA-helicases.

Interestingly, in the case of mutation in the uORF start codon, LNA3 exhibits approximately the same inhibitory properties as the other ASOs (Figure 6D). Thus, the low efficiency of translation inhibition by LNA3 in the WT reporter is likely attributable to uORF translation. Hair_short might be suggested to enhance uORF translation, so LNA3, by unwinding it, would be expected to reduce uORF translation and correspondingly increase translation of the CDS, thereby yielding the observed low apparent efficacy of LNA3 due to mixed positive and negative effects. However, this explanation is unlikely. First, by unwinding the endogenous hairpin (T_m_ close to 60 °C), LNA3 forms an even more stable mRNA–LNA3 duplex (predicted T_m_ 82 °C, Table 1) closer to the uORF start codon, which could even enhance uORF translation and, relative to other ASOs, more effectively inhibit translation of the CDS. Second, although LNA4, like LNA5 and LNA6, masks the CDS start codon, its effects on translation should be different due to its partial overlap with Hair_short. Because none of these outcomes is observed, we suppose that both the endogenous hairpin and the mRNA–LNA3 duplex are insufficiently stable to increase uORF translation efficiency at that distance (>15 nt) from the uORF start codon. Moreover, enhancement of translation by a downstream hairpin has been reported primarily for start codons in a weak context [61,62,68], whereas in DDX23, the context is strong: A at –3 and G at +4 positions (Figure 1) considering the optimal Kozak context [69].

A more plausible explanation is that the ribosome translating uORF impedes LNA3 binding to the 5′ UTR and/or promotes the dissociation of already bound LNA3, which accounts for its low efficacy as a translation inhibitor. A similar effect is known for ASOs targeting CDS [65]; however, to our knowledge, it has not been described for uORFs. This model also explains the concentration-independent effects of LNA3 on translation of the wild-type construct (Figure 6C). Our explanation is indirectly supported by ribosome profiling data (Figure 1C), indicating high ribosome occupancy on the uORF of endogenous *DDX23* mRNA, higher than that on the CDS. This suggests that the LNA3 binding site is particularly well shielded by translating ribosomes.

Thus, our study of LNA-ASOs suggests a new hypothesis/design principle for ASOs inhibiting translation: an ASO should not target an efficiently translated uORF because the high ribosome density in this region will preclude effective binding to the 5′ UTR. Although this principle was uncovered in the context of the *DDX23* 5′ UTR, its general applicability across other uORF-containing mRNAs remains to be explored. Future works systematically investigating ASO performance in relation to uORF translation efficiency in diverse transcripts will be valuable to solidify this principle. Furthermore, given that neither the unwinding of Hair_short with LNA4 has a different effect compared to LNA5 and LNA6, nor the formation of a more stable mRNA–LNA3 duplex closer to the uORF start codon reduces the efficiency of the CDS translation, we hypothesize that Hair_short does not affect uORF translation efficiency but rather may act as a weak inhibitor of the CDS translation by imposing steric hindrance to PIC scanning along the 5′ UTR or have a role unrelated to translation.

In conclusion, while a subset of the designed LNA-ASOs, particularly those targeting the cap-proximal region (LNA1, LNA2), showed the most potent inhibition, the comprehensive structural analysis of the *DDX23* 5′ UTR was critical for the rational design and interpretation of these results. Firstly, it demonstrated that the putative rG4-forming sequences were not stable regulatory elements, steering our focus towards characterized hairpin structures. Secondly, the experimentally confirmed stability of these hairpins validated our use of high-affinity, fully LNA-modified ASOs, explaining the general efficacy observed across multiple targets. Finally, the structural and functional map was essential for deciphering the distinct behavior of LNA3. Its low efficacy in the wild-type context could be attributed to steric hindrance from ribosomes actively translating the upstream uORF—a insight that would have been impossible without the integrated bioinformatic, structural, and functional data. Thus, the value of the structural work lies not in discriminating between similarly effective LNAs but in providing a mechanistic framework that explains both the general success and the specific exceptions, leading to a new design principle for translation-regulating ASOs.

## 3. Materials and Methods

### 3.1. Oligonucleotide Synthesis and Sample Preparation

Unmodified RNAs were synthesized according to the optimized synthetic protocol on an automated ASM-800 DNA/RNA synthesizer (Biosset, Novosibirsk, Russia). For detritylation, capping, and oxidation, 3% solution of dichloroacetic acid in CH_2_Cl_2_, propionic anhydride and *N*-methylimidazole, and iodine solution in tetrahydrofuran: water: pyridine mixture, respectively, were used. At the condensation stage, 0.1 M solutions of protected 2′-*O*-TBDMS-ribonucleotide 3′-phosphoramidites (ChemGen, Wilmington, MA, USA) in absolute CH_3_CN and 0.25 M solution of activator 42 (Sigma-Aldrich, St. Louis, MO, USA) in absolute CH_3_CN were used. The time of condensation was 7 min. The last 5′-*O*-dimetoxytrityl group was removed, and oligonucleotides were deprotected and cleaved from CPG support by treatment with AMA (40% methylamine: 30% ammonium, 1:1) for 2 h at room temperature with subsequent treatment with NMP:TEA:TEA·3HF mixture for 1.5 h at 65 °C. After the addition of ethoxytrimethylsilane (Sigma-Aldrich, St. Louis, MO, USA), precipitation with ethyl ether was carried out. All RNAs were isolated by preparative denaturating 15% PAGE. The RNA homogeneity was analyzed by analytical gel electrophoresis in denaturating 15% PAGE. Fully LNA-modified ASOs were purchased from Lumiprobe (Moscow, Russia).

RNA solutions in 20 mM sodium phosphate buffer supplemented with 140 mM KCl (K^+^-buffer) or LiCl (Li^+^-buffer), pH 7.4, were annealed rapidly (heated to 90 °C and then snap-cooled on ice) to facilitate intramolecular folding. Thioflavin T (ThT) and pyridostatin (PDS) were obtained from Sigma-Aldrich (St. Louis, MO, USA).

### 3.2. Thioflavin T (ThT) Light-Up Assays

Experiments were conducted using 384-well U-Bottom White Polystyrene microplates (CORNING, Corning, NY, USA). Annealed RNA samples in K^+^-buffer and ThT were mixed to final concentrations of 2 µM and 1 µM, respectively, and incubated for 15 min. Measurements were performed at 25 °C. Fluorescence emission spectra were collected from 470 nm to 650 nm with excitation at 440 nm using a M200 PRO Tecan microplate reader (Tecan Group Ltd., Männedorf, Switzerland). Fluorescence intensities at 485 nm were used for data analysis.

### 3.3. Circular Dichroism (CD) and UV Spectroscopy and Melting

CD and UV measurements were performed using a Chirascan spectropolarimeter (Applied Photophysics, Leatherhead, UK) with a 10 mm optical path quartz cuvette. CD spectra of annealed 2 µM RNA samples in K^+^- or Li^+^-buffer were recorded at 10 °C from 230 nm to 300 nm. TDS spectra were obtained by subtracting the absorption spectra in the range 230 nm to 320 nm recorded at 90 °C from those recorded at 10 °C. For the experiments with PDS, annealed 2 µM RNA samples in K^+^-buffer were supplemented with PDS (2.5 mM stock solution in DMSO) to a 2 µM final concentration or an equivalent DMSO volume. Each reported spectrum represents an average of 2 scans. Melting/annealing experiments were conducted with a heating/cooling rate of 1 °C/min from 20 °C to 90 °C, monitoring changes at 265 nm (CD melting) or 295 nm (UV melting).

### 3.4. NMR Experiments

Samples was prepared in DEPC-treated water (100 μM RNA oligonucleotide, 25 mM PBS, 100 mM KCl, 90:10 H_2_O/D_2_O, pH 7.0) and annealed rapidly by heating at 90 °C for 5 min and then snap-cooling on ice for 10 min. NMR spectra were acquired at 25 °C on a Bruker Avance III 600 MHz spectrometer (Bruker, Mannheim, Germany) equipped with a triple resonance cryogenic probe and Bruker Topspin 3.2 software. ^1^H spectra were recorded using the standard zgesgp pulse sequence with water suppression at 4.7 ppm, 16 dummy scans and 1024 to 4096 scans. The pre-scan delay was set to 1 sec. FIDs were processed with an exponential window function and line broadening of 5.0 Hz.

### 3.5. Cell Culturing

The HEK293T cells (ATCC) were cultivated in DMEM/F12 medium (Servicebio, Wuhan, China) supplemented with L-alanyl-L-glutamine (Himedia, Mumbai, India), 10% fetal bovine serum (Himedia, Mumbai, India), 100 units/mL penicillin, and 100 μg/mL streptomycin (Himedia) at 37 °C in 5% CO_2_ atmosphere. Cells were routinely tested for the absence of mycoplasma using a MycoReport kit (Evrogen, Moscow, Russia).

### 3.6. Plasmid Constructs

The plasmid for the dual luciferase assay, containing firefly (Fluc) and *Renilla* (Rluc) under different promoters, was made from the pGL3-Rluc vector (a kind gift from Radik R. Shafikov, Lomonosov Moscow State University, Moscow, Russia). The vector backbone was amplified using Q5 High-Fidelity DNA Polymerase (NEB, Ipswich, MA, USA) with primers 5′-AGACGAGTTGGCACGTCTCAGGAGAAGACGCCAAAAACATAAAG-3′ and 5′-TAAGAGCTCGGTACCTATCGATAGAGAAATGTTCTGGCAC-3′, and the EF-1α core promoter was amplified from the lentiCas9-Blast plasmid (Addgene #52962, Watertown, MA, USA) using primers 5′-AACATTTCTCTATCGATAGGTACCGAGCTCTTAGGGCAGAGCGCACATCG-3′ and 5′-TTCTCCTGAGACGTGCCAACTCGTCTCGACCTAACCGGTCCTGTGTTCTG-3′. The products were assembled into the reporter vector using NEBuilder HiFi DNA Assembly Master Mix (NEB, Ipswich, MA, USA), introducing two BsmBI sites after the EF-1α core promoter and immediately before the Fluc ORF without the AUG codon. The 5′ end of the *DDX23* transcript, including 20 codons of CDS, was amplified from cDNA, obtained by the reverse transcriptase Magnus and oligo(dT)_15_ primer (Evrogen, Moscow, Russia), using primers 5′-GAACCTCGTCTCTAGGTTTCATCTCCGCGACCAG-3′ and 5′-TCAATGCGTCTCACTCCCCTTTCCTCCTTGGAAGGT-3′. The PCR product was cloned into the Esp3I sites of the reporter vector using Esp3I (Thermo Fisher, Waltham, MA, USA) and T4 ligase (Evrogen, Moscow, Russia). The mutant with an inactivated uORF start codon (no uORF) was obtained by overlap extension PCR of the wild-type sequence using primers 5′-GAAAGAAGGCGACGGCTCCGC-3′ and 5′-AGCCGTCGCCTTCTTTCC-3′. Full reporter sequences are available in the Supplementary Materials.

### 3.7. Transfection

HEK293T cells were transfected with plasmids using the Lipofectamine 3000 Transfection Reagent Kit (Invitrogen, Waltham, MA, USA) in 96-well format according to the manufacturer’s protocol. In brief, 24,000 cells per well were seeded overnight in 200 µL of the medium for plasmid transfection or 100 µL of the medium for mRNA transfection. Then, 50 ng of plasmid DNA or a mixture containing 49 ng of Fluc reporter mRNA, 1 ng of Rluc control mRNA, and 10 pmol of LNA ASO, as well as 0.2 µL of Lipofectamine 3000 Transfection Reagent per well were used. If a treatment was required, the medium was replaced with a substance-supplemented medium prior to adding the transfection mixtures. The cells were harvested 4 h after mRNA transfection and 24 h after plasmid transfection for luciferase assays.

### 3.8. In Vitro Transcription

Fluc reporter constructs were amplified (Q5 High-Fidelity DNA Polymerase, NEB) from plasmid reporters using primers 5′-GAGTACTTAATACGACTCACTATAGATCTCCGCGACCAGGAAACG-3′ and 5′-TTTTTTTTTTTTTTTTTTTTTTTTTTTTTTTTTTTTTTTTTTTTTTTTTTCCGCCCCGACTCTAGAATTACACGG-3′, and the Rluc control was amplified from pGL4.70[hRluc] (Promega, Madison, WI, USA) using primers 5′-GAGTACTTAATACGACTCACTATAGTGGTTCTTTCCGCCTCAGGCCACCATGGCTTCCAAG-3′ and 5′-TTTTTTTTTTTTTTTTTTTTTTTTTTTTTTTTTTTTTTTTTTTTTTTTTTCTCTAGAATTACTGCTCGTTCTTCAGCACG-3′ to add the T7 promoter and a poly(A) tail consisting of 50 adenosine bases. Capped mRNA was synthesized from these PCR products and treated with TURBO DNase using a mMESSAGE mMACHINE T7 Transcription Kit (Invitrogen). The mRNA was subsequently purified with a CleanRNA Standard Kit (Evrogen, Moscow, Russia), and its integrity was assessed by denaturing urea polyacrylamide gel electrophoresis. RNA concentrations were measured using a Qubit RNA HS Assay Kit (Invitrogen).

### 3.9. Luciferase Assay

Luciferase activities were measured using a TransDetect Double-Luciferase Reporter Assay Kit (TransGen Biotech, Beijing, China) according to manufacturer protocol in a 96-well format using Victor X5 plate reader (Perkin Elmer, Shelton, CT, USA).

### 3.10. Visualization of Ribosome Profiling

Aggregate ribosome profiling data were accessed using the RiboCrypt browser for ENST00000308025 in RFP modality for all merged *Homo sapiens* datasets. The link is available below.

https://ribocrypt.org/?dff=all_merged-Homo_sapiens&gene=DDX23-ENSG00000174243&tx=ENST00000308025&library=RFP&frames_type=columns&kmer=1&log_scale=FALSE&log_scale_protein=FALSE&extendLeaders=0&extendTrailers=0&viewMode=FALSE&other_tx=FALSE&add_uorfs=FALSE&add_translon=FALSE&genomic_region=&zoom_range=0:101&customSequence=&phyloP=FALSE&summary_track=FALSE&summary_track_type=lines&collapsed_introns_width=30&collapsed_introns=FALSE&go=TRUE#browser (accessed on 22 September 2025).

### 3.11. Statistical Analysis

The samples were analyzed using the Shapiro–Wilk test, indicating a normal distribution at *p* > 0.05. Statistical analysis was performed using GraphPad Prism 10.3.1 software (GraphPad Software, San Diego, CA, USA).

## 4. Conclusions

Using fluorescence, CD, UV, and ^1^H NMR spectroscopies, two structured elements were identified within *DDX23* 5′ UTR mRNA coding for the full-length protein and then, together with the sequences covering start codons, targeted with LNA-modified ASOs. The study yields several significant outcomes. First, it suggests a new principle for the design of translation-inhibiting ASOs: avoid targeting efficiently translated uORFs because their high ribosome density blocks ASO binding. Second, there are several elements in *DDX23* mRNA 5′ UTR relevant to its translation. The first one is uORF, which represses CDS translation, and the others are two structured elements within 44–64 nt and 76–96 nt. The hairpin Hair_short within 44–64 nt likely inhibits translation of DDX23 mRNA blocking PIC scanning, without interference with uORF translation, the 76–96 nt element may have a similar influence. ASOs targeting the 5′ proximal region of mRNA are effective translation inhibitors, and the proposed mechanism involves impaired PIC loading. To carefully modulate uORF translation, thermal stability of the ASO-RNA duplex formed should be carefully tuned to avoid creating a roadblock for translation machinery more powerful than endogenous structures. These findings provide valuable insights for developing more effective ASOs targeting the main *DDX23* transcript, and mRNA in general, and regulating its translation for potential anticancer applications.

## Figures and Tables

**Table 1:** LNA-modified ASOs targeting the uORF and CDS start codons as well as the putative regulatory elements in *DDX23* 5′ UTR.

Code	Sequence 5′→3′	Target Region	LNA/RNA Duplex T_m_, °C	GC, %
LNA1	CATCTTTCCCGTTC	Upstream region to the uORF start codon	134	50
LNA2	GCGGAGCCGTCGCCA	Downstream region to the uORF start codon	98	80
LNA3	AACGCGGCCTCAACG	5′ segment of Hair_short and the uORF stop codon	82	67
LNA4	CACCCTGAGTCTGAA	3′ segment of Hair_short and 5′ segment of G3	120	53
LNA5	TCCTGCCATCACCCT	Central region of G3 and the CDS start codon	123	60
LNA6	AGCCAGCTCTCCTGC	3′ segment of G3	104	67
SCR	CATACGTCTATACGCT	Negative control	-	44

## Data Availability

The original contributions presented in this study are included in the article/Supplementary Materials. Further inquiries can be directed to the corresponding authors.

## Supplementary Materials

The following supporting information can be downloaded at: https://www.mdpi.com/article/10.3390/ijms262211047/s1.

## Abbreviations

The following abbreviations are used in this manuscript:

ASO	Antisense oligonucleotide
CD	Circular dichroism
CDS	Coding sequence
CH_2_Cl_2_	Methylene chloride
CH_3_CN	Acetonitrile
CPG	Controlled pore glass
DDX23	DEAD-box helicase 23
DEPC	Diethyl pyrocarbonate
DMEM	Dulbecco’s Modified Eagle Medium
DMSO	Dimethyl sulfoxide
dsRNA	Double-stranded RNA
ER	Estrogen receptor
HF	Hydrofluoric acid
LNA	Locked nucleic acid
NMP	N-Methyl-2-pyrrolidone
NMR	Nuclear magnetic resonance
PBS	Phosphate-buffered saline
PDS	Pyridostatin
PIC	Pre-initiation complex
ppm	Parts per million
rG4	RNA G-quadruplex
SCR	Scramble
rTBA	RNA 15-nt thrombin binding aptamer
TBDMS	tert-Butyldimethylsilyl
TDS	Thermal difference spectra
TEA	Triethylamine
ThT	Thioflavin T
T_m_	Melting temperature
uORF	Upstream open reading frame
5′ UTR	5′ Untranslated region
UV	Ultraviolet
