# Supplementary material for: Structural Characterization of DDX23 5′ UTR Regulatory Elements and Their Targeting by LNA-Modified Antisense Oligonucleotides
Source: Int J Mol Sci. 2025 Nov 14;26(22):11047. doi: 10.3390/ijms262211047 (PMC12652805; doi:10.3390/ijms262211047)
Supplement: Supplementary file 1 [file ijms-26-11047-s001.zip › ijms-3943294-supplementary.pdf]

Structural Characterization of *DDX23* 5′ UTR Regulatory Elements and Their Targeting by LNA-Modified Antisense Oligonucleotides

Polina Kamzeeva [1,†], Nikita Shepelev [1,2,†], Veronika Zabbarova [2], Vladimir Brylev [1], Alexey Chistov [1], Dmitriy Ryazantsev [1], Erik Kot [1], Darya Novopashina [3], Maria Rubtsova [1,2,*] and Andrey Aralov [1,4,5,*]

[1] Shemyakin-Ovchinnikov Institute of Bioorganic Chemistry, The Russian Academy of Sciences, 117437 Moscow, Russia
[2] Chemistry Department, Lomonosov Moscow State University, 119234 Moscow, Russia
[3] Institute of Chemical Biology and Fundamental Medicine, Siberian Branch of the Russian Academy of Sciences, 630090 Novosibirsk, Russia
[4] Lopukhin Federal Research and Clinical Center of Physical-Chemical Medicine, Federal Medical Biological Agency, 119435 Moscow, Russia
[5] Educational Resource Center for Cellular Technologies, RUDN University, 117198 Moscow, Russia
* Correspondence: mprubtsova@gmail.com (M.R.); baruh238@mail.ru (A.A.)
† These authors contributed equally to this work.

Table S1. RNA oligonucleotides used in this study.

Code		Sequence 5'→3'
G1		r(CCAGGAAACGGGAAAGAUGGCGACGGCUC)
mutG1		r(CCAGUAAACGUGAAAGAUUGCGACGUCUC)
G2		r(UGAGGCCGCGUUGGGCGGUUCAGACUCAGGGUG)
mutG2		r(UGAGUCCGCGUUGUGCGAUUCAGACUCAGAGUG)
G3		r(UCAGGGUGAUGGCAGGAGAGCUGGCUG)
mutG3		r(UCAGUGUGAUGUCAGUAGAGCUGUCUG)
h_mutG3		r(UCAGGGAGAAGGCAGGAGAGCAGGAUG)
Hair_long		r(CGUUGAGGCCGCGUUGGGCGGUUCAGACUCAGGG), -12.7 kkal/mol[a]
Hair_short		r(GGCCGCGUUGGGCGGUUCAGAC), -9.1 kkal/mol[a]
rTBA		r(GGUUGGUGUGGUUGG)
BON	BON1	r(AUAAAAUAAAAAAUUACACUGG)
	BON2	r(CCAGUGUAAUUUUUUAUUUUAU)

[a] – based on ViennaRNAfold computational analysis [1,2]

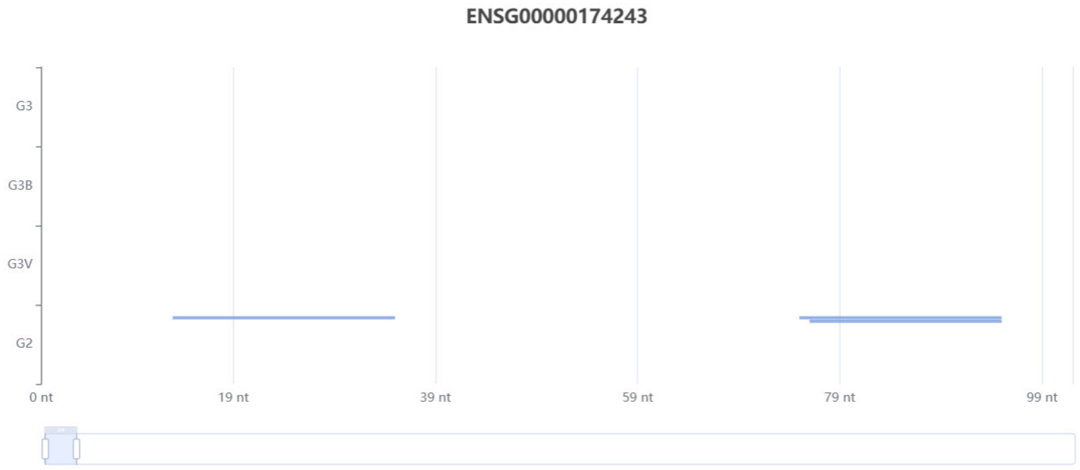

Figure S1. rG4-forming sequences in accordance with G4Atlas database

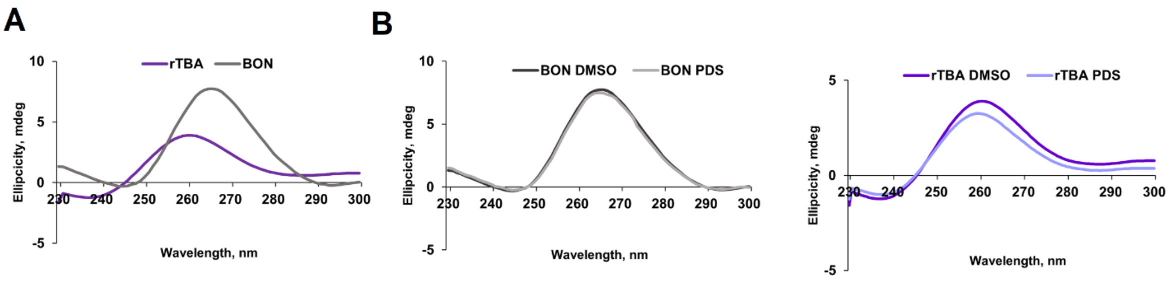

Figure S2. CD spectra of rTBA and BON (A) and the effect of the addition of 1 eq. of PDS (B). Conditions: 2 μM RNA, 20 mM sodium phosphate buffer, pH 7.4, 140 mM KCl.

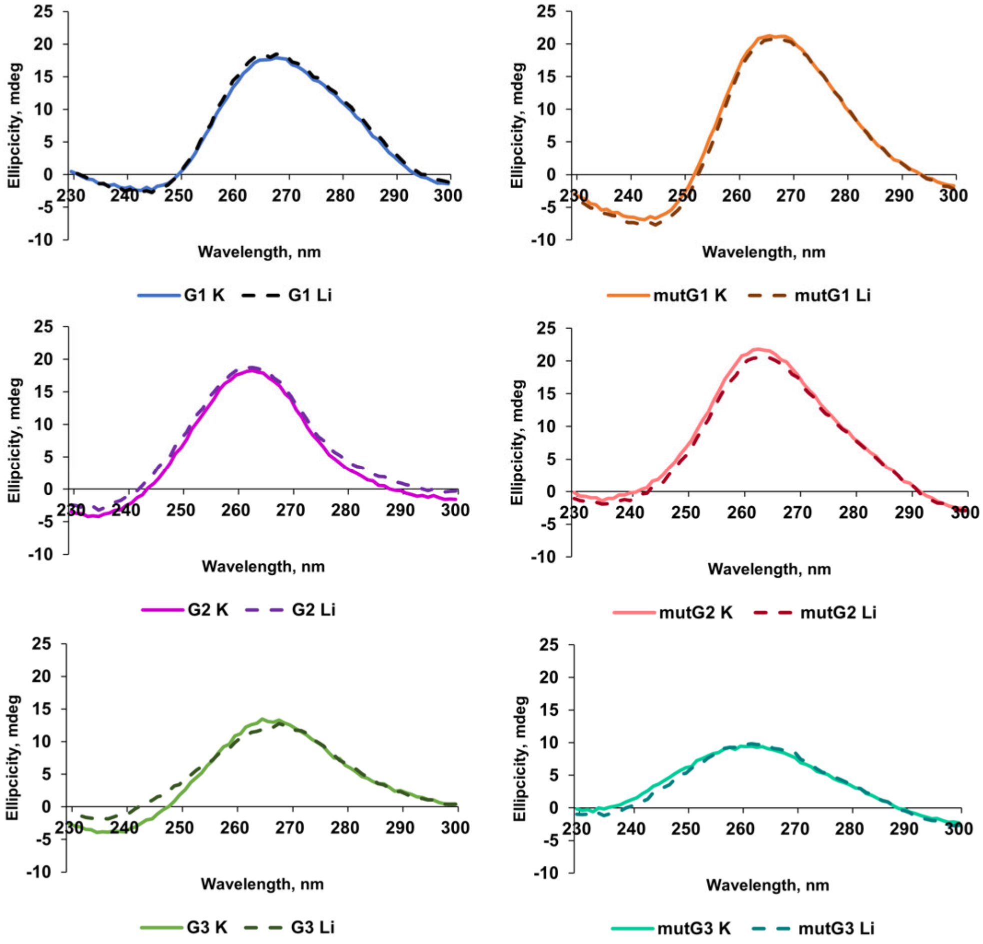

Figure S3. CD spectra of putative rG4-forming sequences G1, G2 and G3 and their G-to-U mutants in K^+- and Li^+-containing buffers. Conditions: 2 μM RNA, 20 mM sodium phosphate buffer, pH 7.4, 140 mM KCl (K^+-containing buffers) or LiCl (Li^+-containing buffer).

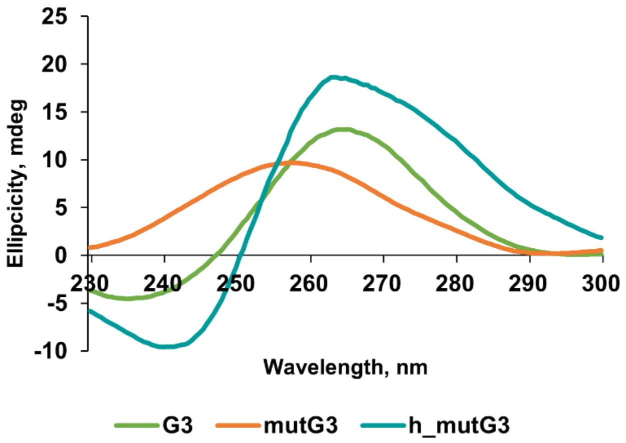

Figure S4. CD spectra of G3, h_mutG3 and mutG3 ONs. Conditions: 2 μM RNA, 20 mM sodium phosphate buffer, pH 7.4, 140 mM KCl.

Luciferase reporter (pGL3-EFS-Rluc-v2) sequence with key elements highlighted:

EF-1α core promoter

Fluc

SV40 poly(A) signal

SV40 promoter

Rluc

TACCATGATAAGTAAGTAATATTAAGGTACGGGAGGTACTTGGAGCGGCCGCAATAAA
ATATCTTTATTTTCATTACATCTGTGTGTTGGTTTTTTGTGTGAATCGATAGTACTAACAT
ACGCTCTCCATCAAAACAAAACGAAACAAAACAAACTAGCAAAATAGGCTGTCCCCA
GTGCAAGTGCAGGTGCCAGAACATTTCTCTATCGATAGGTACCGAGCTCTTAGGGCAGA
GCGCACATCGCCCACAGTCCCCGAGAAGTTGGGGGGAGGGGTCGGCAATTGATCCGGT
GCCTAGAGAAGGTGGCGCGGGGTAAACTGGGAAAGTGATGTCGTGTACTGGCTCCGCC
TTTTTCCCGAGGGTGGGGGAGAACCGTATATAAGTGCAGTAGTCGCCGTGAACGTTCTT
TTTCGCAACGGGTTTGCCGCCAGAACACAG**(5′ UTR+CDS codons)**GAAGACGCCAAAAA
CATAAAGAAAGGCCCGGCGCCATTCTATCCGCTGGAAGATGGAACCGCTGGAGAGCAA
CTGCATAAGGCTATGAAGAGATACGCCCTGGTTCCTGGAACAATTGCTTTTACAGATGC
ACATATCGAGGTGGACATCACTTACGCTGAGTACTTCGAAATGTCCGTTCGGTTGGCAG
AAGCTATGAAACGATATGGGCTGAATACAAATCACAGAATCGTCGTATGCAGTGAAAA
CTCTCTTCAATTCTTTATGCCGGTGTTGGGCGCGTTATTTATCGGAGTTGCAGTTGCGCCC
GCGAACGACATTTATAATGAACGTGAATTGCTCAACAGTATGGGCATTTCGCAGCCTAC
CGTGGTGTTCGTTTCCAAAAAGGGGTTGCAAAAAATTTTGAACGTGCAAAAAAAGCTC
CCAATCATCCAAAAAATTATTATCATGGATTCTAAAACGGATTACCAGGGATTTCAGTC
GATGTACACGTTCGTCACATCTCATCTACCTCCCGGTTTTAATGAATACGATTTTGTGCC
AGAGTCCTTCGATAGGGACAAGACAATTGCACTGATCATGAACTCCTCTGGATCTACTG
GTCTGCCTAAAGGTGTCGCTCTGCCTCATAGAACTGCCTGCGTGAGATTCTCGCATGCC
AGAGATCCTATTTTTGGCAATCAAATCATTCCGGATACTGCGATTTTAAGTGTTGTTCCA

TTCCATCACGGTTTTGGAATGTTTACTACACTCGGATATTTGATATGTGGATTTCGAGTC
GTCTTAATGTATAGATTTGAAGAAGAGCTGTTTCTGAGGAGCCTTCAGGATTACAAGAT
TCAAAGTGCGCTGCTGGTGCCAACCCTATTCTCCTTCTTCGCCAAAAGCACTCTGATTGA
CAAATACGATTTATCTAATTTACACGAAATTGCTTCTGGTGGCGCTCCCCTCTCTAAGGA
AGTCGGGGAAGCGGTTGCCAAGAGGTTCCATCTGCCAGGTATCAGGCAAGGATATGGG
CTCACTGAGACTACATCAGCTATTCTGATTACACCCGAGGGGGATGATAAACCGGGCG
CGGTCGGTAAAGTTGTTCCATTTTTTGAAGCGAAGGTTGTGGATCTGGATACCGGGAAA
ACGCTGGGCGTTAATCAAAGAGGCGAACTGTGTGTGAGAGGTCCTATGATTATGTCCGG
TTATGTAAACAATCCGGAAGCGACCAACGCCTTGATTGACAAGGATGGATGGCTACAT
TCTGGAGACATAGCTTACTGGGACGAAGACGAACACTTCTTCATCGTTGACCGCCTGAA
GTCTCTGATTAAGTACAAAGGCTATCAGGTGGCTCCCGCTGAATTGGAATCCATCTTGC
TCCAACACCCCAACATCTTCGACGCAGGTGTCGCAGGTCTTCCCGACGATGACGCCGGT
GAACTTCCCGCCGCCGTTGTTGTTTTGGAGCACGGAAAGACGATGACGGAAAAAGAGA
TCGTGGATTACGTCGCCAGTCAAGTAACAACCGCGAAAAAGTTGCGCGGAGGAGTTGT
GTTTGTGGACGAAGTACCGAAAGGTCTTACCGGAAAACTCGACGCAAGAAAAATCAGA
GAGATCCTCATAAAGGCCAAGAAGGGCGGAAAGATCGCCGTGTAATTCTAGAGTCGGG
GCGGCCGGCCGCTTCGAGCAGACATGATAAGATACATTGATGAGTTTGGACAAACCAC
AACTAGAATGCAGTGAAAAAAATGCTTTATTTGTGAAATTTGTGATGCTATTGCTTTATT
TGTAACCATTATAAGCTGCAATAAACAAGTTGGGCTCGAGATCTGCGATCTGCATCTCA
ATTAGTCAGCAACCATAGTCCCGCCCCTAACTCCGCCCATCCCGCCCCTAACTCCGCCC
AGTTCCGCCCATTCTCCGCCCCATCGCTGACTAATTTTTTTTATTTATGCAGAGGCCGAG
GCCGCCTCGGCCTCTGAGCTATTCCAGAAGTAGTGAGGAGGCTTTTTTGGAGGCCTAGG
CTTTTGCAAAAAGCTGAAGTTGGTCGTGAGGCACTGGGCAGGTAAGTATCAAGGTTAC
AAGACAGGTTTAAGGAGACCAATAGAAACTGGGCTTGTCGAGACAGAGAAGACTCTTG
CGTTTCTGATAGGCACCTATTGGTCTTACTGACATCCACTTTGCCTTTCTCTCCACAGGTG
TCCACTCCCAGTTCAATTACAGCTCTTAAGGCTAGAGTACTTAATACGACTCACTATAG
GCTAGGCTTGGCATTCCGGTACTGAATTGATATCAAGCTCTAGCCACCATGACTTCGAA
AGTTTATGATCCAGAACAAAGGAAACGGATGATAACTGGTCCGCAGTGGTGGGCCAGA
TGTAAACAAATGAATGTTCTTGATTCATTTATTAATTATTATGATTCAGAAAAACATGCA
GAAAATGCTGTTATTTTTTTACATGGTAACGCGGCCTCTTCTTATTTATGGCGACATGTTG
TGCCACATATTGAGCCAGTAGCGCGGTGTATTATACCAGACCTTATTGGTATGGGCAAA
TCAGGCAAATCTGGTAATGGTTCTTATAGGTTACTTGATCATTACAAATATCTTACTGCA
TGGTTTGAACTTCTTAATTTACCAAAGAAGATCATTTTTGTCGGCCATGATTGGGGTGCT
TGTTTGGCATTTCATTATAGCTATGAGCATCAAGATAAGATCAAAGCAATAGTTCACGC
TGAAAGTGTAGTAGATGTGATTGAATCATGGGATGAATGGCCTGATATTGAAGAAGAT
ATTGCGTTGATCAAATCTGAAGAAGGAGAAAAAATGGTTTTGGAGAATAACTTCTTCGT
GGAAACCATGTTGCCATCAAAAATCATGAGAAAGTTAGAACCAGAAGAATTTGCAGCA
TATCTTGAACCATTCAAAGAGAAAGGTGAAGTTCGTCGTCCAACATTATCATGGCCTCG
TGAAATCCCGTTAGTAAAAGGTGGTAAACCTGACGTTGTACAAATTGTTAGGAATTATA
ATGCTTATCTACGTGCAAGTGATGATTTACCAAAAATGTTTATTGAATCGGACCCAGGA
TTCTTTTCCAATGCTATTGTTGAAGGTGCCAAGAAGTTTCCTAATACTGAATTTGTCAAA
GTAAAAGGTCTTCATTTTTCGCAAGAAGATGCACCTGATGAAATGGAAAATATATCA
AATCGTTCGTTGAGCGAGTTCTCAAAAATGAACAATAATTCTAGAGTCGGGGCGGCCG
GCCGCTTCGAGCAGACATGATAAGATACATTGATGAGTTTGGACAAACCACAACTAGA
ATGCAGTGAAAAAAATGCTTTATTTGTGAAATTTGTGATGCTATTGCTTTATTTGTAACC

ATTATAAGCTGCAATAAACAAGTTAACAACAACAATTGCATTCATTTTATGTTTCAGGTT
CAGGGGGAGGTGTGGGAGGTTTTTTAAAGCAAGTAAAACCTCTACAAATGTGGTAAAA
TCGATAAGGATCTGAACGATGGAGCGGAGAATGGGCGGAACTGGGCGGAGTTAGGGG
CGGGATGGGCGGAGTTAGGGGCGGGACTATGGTTGCTGACTAATTGAGATGCATGCTTT
GCATACTTCTGCCTGCTGGGGAGCCTGGGGACTTTCCACACCTGGTTGCTGACTAATTGA
GATGCATGCTTTGCATACTTCTGCCTGCTGGGGAGCCTGGGGACTTTCCACACCCTAACT
GACACACATTCCACAGCGGATCCGTCGACCGATGCCCTTGAGAGCCTTCAACCCAGTC
AGCTCCTTCCGGTGGGCGCGGGGCATGACTATCGTCGCCGCACTTATGACTGTCTTCTTT
ATCATGCAACTCGTAGGACAGGTGCCGGCAGCGCTCTTCCGCTTCCTCGCTCACTGACT
CGCTGCGCTCGGTCGTTCGGCTGCGGCGAGCGGTATCAGCTCACTCAAAGGCGGTAATA
CGGTTATCCACAGAATCAGGGGATAACGCAGGAAAGAACATGTGAGCAAAAGGCCAG
CAAAAGGCCAGGAACCGTAAAAAGGCCGCGTTGCTGGCGTTTTTCCATAGGCTCCGCC
CCCCTGACGAGCATCACAAAAATCGACGCTCAAGTCAGAGGTGGCGAAACCCGACAG
GACTATAAAGATACCAGGCGTTTCCCCCTGGAAGCTCCCTCGTGCGCTCTCCTGTTCCG
ACCCTGCCGCTTACCGGATACCTGTCCGCCTTTCTCCCTTCGGGAAGCGTGGCGCTTTCT
CATAGCTCACGCTGTAGGTATCTCAGTTCGGTGTAGGTCGTTCGCTCCAAGCTGGGCTGT
GTGCACGAACCCCCCGTTCAGCCCGACCGCTGCGCCTTATCCGGTAACTATCGTCTTGA
GTCCAACCCGGTAAGACACGACTTATCGCCACTGGCAGCAGCCACTGGTAACAGGATT
AGCAGAGCGAGGTATGTAGGCGGTGCTACAGAGTTCTTGAAGTGGTGGCCTAACTACG
GCTACACTAGAAGAACAGTATTTGGTATCTGCGCTCTGCTGAAGCCAGTTACCTTCGGA
AAAAGAGTTGGTAGCTCTTGATCCGGCAAACAAACCACCGCTGGTAGCGGTGGTTTTTT
TGTTTGCAAGCAGCAGATTACGCGCAGAAAAAAAGGATCTCAAGAAGATCCTTTGATC
TTTTCTACGGGGTCTGACGCTCAGTGGAACGAAAACTCACGTTAAGGGATTTTGGTCAT
GAGATTATCAAAAAGGATCTTCACCTAGATCCTTTTAAATTAAAAATGAAGTTTTAAAT
CAATCTAAAGTATATATGAGTAAACTTGGTCTGACAGTTACCAATGCTTAATCAGTGAG
GCACCTATCTCAGCGATCTGTCTATTTCGTTCATCCATAGTTGCCTGACTCCCCGTCGTGT
AGATAACTACGATACGGGAGGGCTTACCATCTGGCCCCAGTGCTGCAATGATACCGCG
AGACCCACGCTCACCGGCTCCAGATTTATCAGCAATAAACCAGCCAGCCGGAAGGGCC
GAGCGCAGAAGTGGTCCTGCAACTTTATCCGCCTCCATCCAGTCTATTAATTGTTGCCGG
GAAGCTAGAGTAAGTAGTTCGCCAGTTAATAGTTTGCGCAACGTTGTTGCCATTGCTAC
AGGCATCGTGGTGTCACGCTCGTCGTTTGGTATGGCTTCATTCAGCTCCGGTTCCCAACG
ATCAAGGCGAGTTACATGATCCCCCATGTTGTGCAAAAAAGCGGTTAGCTCCTTCGGTC
CTCCGATCGTTGTCAGAAGTAAGTTGGCCGCAGTGTTATCACTCATGGTTATGGCAGCA
CTGCATAATTCTCTTACTGTCATGCCATCCGTAAGATGCTTTTCTGTGACTGGTGAGTACT
CAACCAAGTCATTCTGAGAATAGTGTATGCGGCGACCGAGTTGCTCTTGCCCGGCGTCA
ATACGGGATAATACCGCGCCACATAGCAGAACTTTAAAAGTGCTCATCATTGGAAAAC
GTTCTTCGGGGCGAAAACTCTCAAGGATCTTACCGCTGTTGAGATCCAGTTCGATGTAA
CCCACTCGTGCACCCAACTGATCTTCAGCATCTTTTACTTTCACCAGCGTTTCTGGGTGA
GCAAAAACAGGAAGGCAAAATGCCGCAAAAAAGGGAATAAGGGCGACACGGAAATG
TTGAATACTCATACTCTTCCTTTTTCAATATTATTGAAGCATTTATCAGGGTTATTGTCTC
ATGAGCGGATACATATTTGAATGTATTTAGAAAAATAAACAAATAGGGGTTCCGCGCA
CATTTCCCCGAAAAGTGCCACCTGACGCGCCCTGTAGCGGCGCATTAAGCGCGGCGGG
TGTGGTGGTTACGCGCAGCGTGACCGCTACACTTGCCAGCGCCCTAGCGCCCGCTCCTT
TCGCTTTCTTCCCTTCCTTTCTCGCCACGTTCGCCGGCTTTCCCCGTCAAGCTCTAAATCG
GGGGCTCCCTTTAGGGTTCCGATTTAGTGCTTTACGGCACCTCGACCCCAAAAAACTTG

ATTAGGGTGATGGTTCACGTAGTGGGCCATCGCCCTGATAGACGGTTTTTCGCCCTTTGA
CGTTGGAGTCCACGTTCTTTAATAGTGGACTCTTGTTCCAAACTGGAACAACACTCAAC
CCTATCTCGGTCTATTCTTTTGATTTATAAGGGATTTTGCCGATTTCGGCCTATTGGTTAA
AAAATGAGCTGATTTAACAAAAATTTAACGCGAATTTTAACAAAATATTAACGCTTACA
ATTTGCCATTCGCCATTCAGGCTGCGCAACTGTTGGGAAGGGCGATCGGTGCGGGCCTC
TTCGCTATTACGCCAGCCCAAGC

List of 5′ UTRs used (CDS attached to Fluc is in bold, 5′ UTR is underlined):

Short 5′ UTR

GACCGGTTGTACAGAGACGCGCCACC**ATGGGAGAC**

Wild type 5′ UTR *DDX23*

GACCGGTTAGGTTTCATCTCCGCGACCAGGAAACGGGAAAGATGGCGACGGCTCCGCG
ACGTTGAGGCCGCGTTGGGCGGTTCAGACTCAGGGTG**ATGGCAGGAGAGCTGGCTGA
CAAAAAGGACCGTGATGCATCACCTTCCAAGGAGGAAAGGGGA**

No uORF 5′ UTR *DDX23*

GACCGGTTAGGTTTCATCTCCGCGACCAGGAAACGGGAAAGAAGGCGACGGCTCCGCG
ACGTTGAGGCCGCGTTGGGCGGTTCAGACTCAGGGTG**ATGGCAGGAGAGCTGGCTGA
CAAAAAGGACCGTGATGCATCACCTTCCAAGGAGGAAAGGGGA**

References

[1] Gruber, A.R.; Lorenz, R.; Bernhart, S.H.; Neuböck, R.; Hofacker, I.L. The Vienna RNA Websuite. Nucleic Acids Res. 2008, 36, W70-74, doi:10.1093/nar/gkn188.

[2] Hofacker, I.L. Vienna RNA Secondary Structure Server. Nucleic Acids Res. 2003, 31, 3429–3431, doi:10.1093/nar/gkg599.
